# Protect, Repair, and Regenerate: Towards Restoring Vision in Glaucoma

**DOI:** 10.1007/s40135-020-00259-5

**Published:** 2020-11-20

**Authors:** Lauren K. Wareham, Michael L. Risner, David J. Calkins

**Affiliations:** Department of Ophthalmology and Visual Sciences and the Vanderbilt Eye Institute, Vanderbilt University Medical Center, AA7100 MCN, 1161 21st Ave S., Nashville, TN 37232 USA

**Keywords:** Optic nerve regeneration, Neuroregeneration, Neuroprotection, Glaucoma, Retinal ganglion cell, Neurorepair

## Abstract

**Purpose of Review:**

We summarize recent advances in strategies that aim to restore optic nerve function and vision in glaucoma through protective, reparative, and regenerative avenues.

**Recent Findings:**

Neuroprotection relies on identification of early retinal ganglion cell dysfunction, which could prove challenging in the clinic. Cell replacement therapies show promise in restoring lost vision, but some hurdles remain in restoring visual circuitry in the retina and central connections in the brain.

**Summary:**

Identification and manipulation of intrinsic and extrinsic cellular mechanisms that promote axon regeneration in both resident and transplanted RGCs will drive future advances in vision restoration. Understanding the roles of multiple cell types in the retina that act in concert to promote RGC survival will aid efforts to promote neuronal health and restoration. Effective RGC transplantation, fine tuning axon guidance and growth, and synaptogenesis of transplanted and resident RGCs are still areas that require more research.

## Introduction

**Fig. 1:**
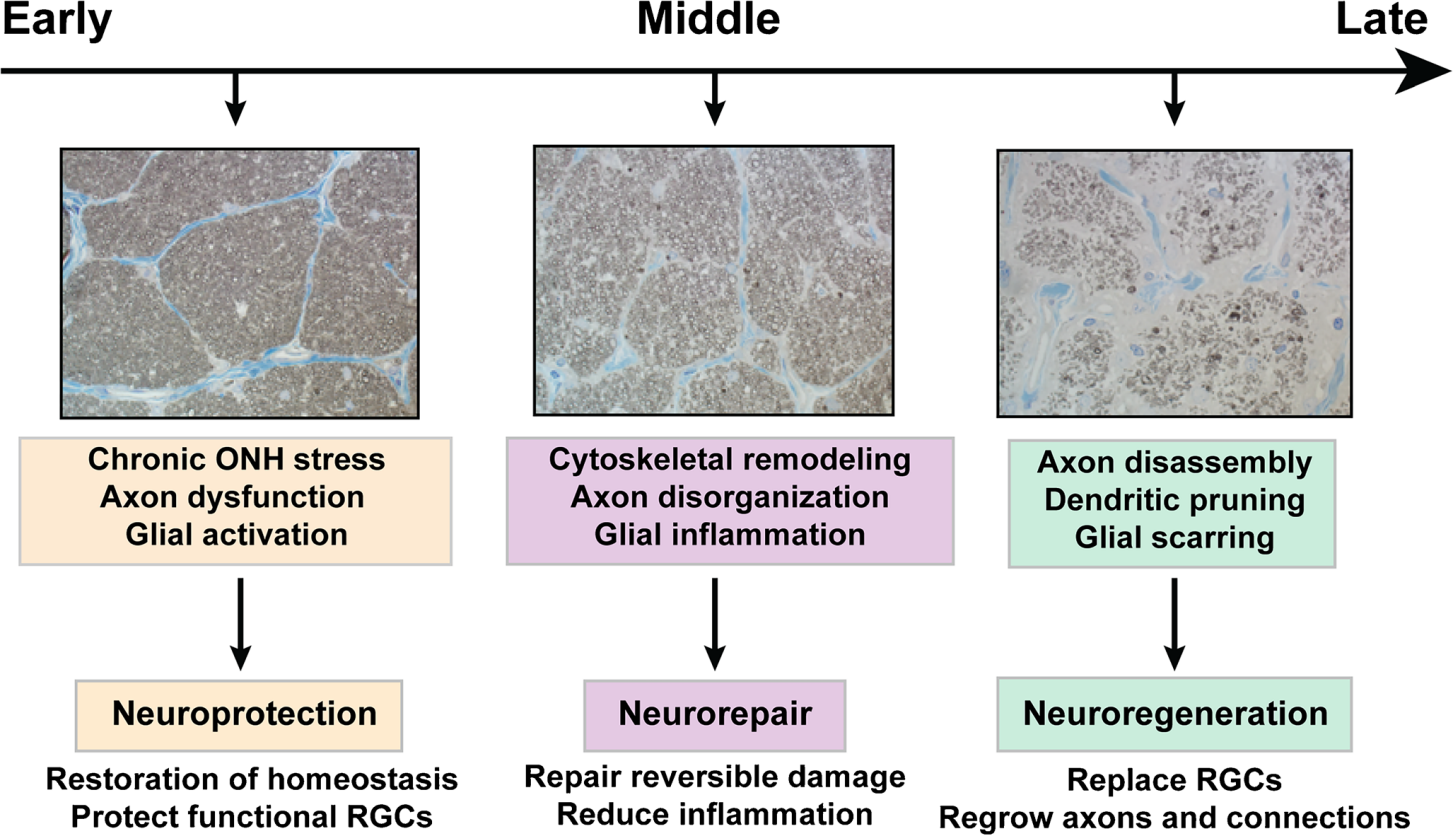
Windows of opportunity for vision restoration in glaucoma. Cross-sections through squirrel monkey optic nerve demonstrate stages of glaucoma. Early in disease progression, stressors at the optic nerve head lead to dysfunction of retinal ganglion cells (RGCs) and their axons and activation of glial cells in the retina and optic nerve. At this stage, neuroprotective strategies aim to restore homeostasis between dysfunctional RGCs and their environment and protect normally functioning RGCs from disease-relevant stress. As glaucoma progresses, early degenerative events include remodeling of cytoskeletal and synaptic structures throughout the RGC projection and increased inflammatory signaling from resident glial cells. At this stage in disease progression, therapies aim to repair reversible damage at the molecular and cellular level and to reduce inflammation to prevent further damage. Later stages include loss of RGCs and their axons with glial scarring in the optic nerve, which is a barrier to axon regeneration. Replacement therapies to restore visual function require RGC replacement and axon regeneration to appropriate central brain targets with remyelination in the optic nerve

Glaucoma is treated as a disease of the anterior segment of the eye, since the only modifiable risk factor is intraocular pressure or IOP. Even so, vision loss in the disease arises from degeneration of the 1.5 million or so retinal ganglion cell (RGC) axons that comprise the optic nerve and form the optic projection to the brain [1]. Even with available hypotensive treatments, many patients with glaucoma continue to progress to sectors of irreversible vision loss and eventual blindness. Since early field defects typically do not affect central vision, glaucoma is often unrecognized by patients until irrevocable damage to neural tissue has already occurred. Therapeutic interventions that target not IOP, but rather the causes of irreversible vision loss should incorporate three avenues corresponding to different points in progression: protection, repair, and regenerate (Fig. 1). Each avenue represents an important therapeutic window in disease progression with distinct characteristics and mechanistic targets. Identifying intrinsic and extrinsic mechanisms that can be leveraged to prevent RGC degeneration, repair dysfunctional cells, and promote regeneration of the optic nerve and projection requires understanding the signatures of IOP-related stress to the neural substrate, compensatory or adaptive responses to that stress, and processes that could be boot-strapped for tissue replacement during these windows. This review will highlight some recent advances in addressing RGC and optic nerve degeneration and will focus on the intrinsic responses that influence RGC axon survival, repair, and regeneration in glaucoma.

## The Optic Nerve Head—a Critical Locus for Degeneration and Potential Regeneration

Sensitivity to IOP in glaucoma involves stress conveyed to RGC axons at the optic nerve head, which induces degenerative events in both distal and proximal structures [1]. In the retina and prelaminar region, RGC axons remain unmyelinated in well-defined bundles (or fascicles) that penetrate a plexus of astrocytes, capillaries, and connective tissues. In leaving the lamina cribrosa, RGC axons finally become myelinated, forming the optic nerve proper. The optic nerve head acts as a biological scaffold, a mesh of connective tissues and cells built to withstand changes in force, stress, and strain due to both fluctuations in IOP and its absolute magnitude [2]. Stress at the ONH conveyed to RGC axons leads to changes in activity, dendritic remodeling, axon atrophy, glial hypertrophy, and eventual glial scar formation.

Stress to RGCs in glaucoma progresses in both directions from the nerve head: retrograde towards the RGC body in the retina and anterograde towards central axonal targets in the brain. Work with transgenic mice demonstrates that the proximal (retrograde) and distal (anterograde) programs progressing on either side of this junction are to some extent independent [3–7]. In the retrograde direction, RGC dendritic arbors experience pruning, with loss of excitatory synapses. In the anterograde direction, active axonal transport from the retina to central brain terminals diminishes early in progression, with subsequent disassembly of the myelinated axon in the nerve and degradation of post-synaptic targets in the brain [8, 9]. Importantly, the unmyelinated axon segment in the retina and proximal nerve head remains intact along with the RGC body long after degeneration of the rest of the axon and pruning of the dendritic arbor. The optic nerve head is therefore a critical locus in the degeneration of RGCs and understanding the underlying mechanisms is key to developing protective, reparative, and regenerative strategies to preserve the optic projection and prevent vision loss in glaucoma.

## Early Glaucoma: Strategies to Protect RGCs

Neuroprotective strategies aim to restore homeostasis between stressed RGCs and their environment in the optic projection to promote survival and protect unaffected RGCs from early progression. Animal studies involving either induced (e.g., microbead occlusion, [10]) or genetic (e.g., DBA/2J and GC-1^−/−^ mice [10–12]) elevations in IOP have proven invaluable in identifying key molecular events in RGC dysfunction, which highlight an early window of neuroprotective opportunity. Ideally, neuroprotective strategies should prevent RGC dysfunction and thus subsequent degeneration while promoting repair or even regeneration of damaged tissue to restore vision, as reviewed elsewhere [13]. While clinical neuroprotective trials have proven difficult [14], therapies involving growth factors may have the potential to achieve those goals.

RGC dendrites are dynamic during development, expanding and contracting in response to environmental stimuli, but become stable when cells reach maturity [15]. Shrinkage of dendritic arbors occurs before complete degeneration of cells; thus, dendritic regeneration is a crucial step in the replenishment of RGC health and function [16, 17••]. An important discovery is that RGCs possess the intrinsic ability to regenerate dendrites after injury; application of exogenous insulin after injury drives dendritic growth via the activation of the mammalian target of rapamycin (mTOR) pathway [17••]. Harnessing mTOR-driven regeneration of RGC dendrites may prove to be a useful neuroprotective strategy in glaucoma treatment in the future.

RGC axons extend a great distance from the retina to multiple termination zones in the brain. In murine models of glaucoma, dysfunctional axon transport is evident early in disease [9]. RGC axons rely on functional axonal transport for a plethora of neurotrophic factors, including brain-derived neurotrophic factor (BDNF), nerve growth factor (NGF), and ciliary neurotrophic factor (CNTF) [18, 19]. In glaucoma, transport of BDNF is compromised after elevations in IOP [20], while levels of BDNF are reduced in serum and tears of glaucoma patients [21, 22]. BDNF promotes the survival and growth of RGCs in vitro [23–25], while intravitreal injection of AAV2-derived BDNF significantly protected RGCs from degeneration following optic nerve transection and also improved optokinetic responses [26•]. Neurotrophin-based therapies have shown some efficacy in promoting RGC survival. NGF protects RGCs from injury in vitro [27, 28], while intravitreal administration of NGF and BDNF delays RGC death after acute optic nerve injury but does not prevent complete degeneration [29–31]. In a small study of patients with glaucoma, NGF eyedrops improved multiple aspects of visual acuity [32]. CNTF has some neuroprotective effects in vivo [33, 34] and is capable of stimulating axonal regeneration [35], which may be partially mediated by release of CNTF by astrocytes [36].

Although preclinical research into the neuroprotective effects of growth factors appears promising, one drawback to recombinant growth factor therapy appears to be the rapid clearance in vivo of exogenous neurotrophins, which are protein-based drugs and are highly sensitive to proteolytic degradation [37, 38]. Advancements in the development and use of viral vectors to overexpress neurotrophins in the retina may help to improve outcomes. Overexpression of BDNA or CNTF using viral vectors increases RGC survival after injury [26, 39, 40•, 41, 42]. Initial phase 1 clinical safety trials have shown that human CNTF is safe for human retinal tissue [43]. Recombinant NGF is in phase 1 clinical trials for glaucoma (Clinicaltrials.gov identifier: NCT02855450) after a previous clinical trial showed that topical delivery was well tolerated by patients [44]. While non-invasive retinal imaging utilizing a fluorescent biomarker of RGC apoptosis is helpful in tracking progression [45], translating preclinical findings into regular use for neuroprotection relies on earlier intervention by detecting more subtle changes in RGC function.

## Cell Replacement Strategies

For patients with severe visual field deficits, indicative of gross RGC degeneration, strategies to replace lost RGCs may be the only way forward to restore vision. Photoreceptor replacement to restore vision provides proof-of-concept that integration of new neurons into the mammalian retina is possible [46–49], albeit without the complication of long-distance projection to multiple targets required of RGCs. Animal studies have already highlighted the challenges associated with transplanting RGCs into mature retinal tissue [50•, 51, 52••, 53–57]. The generation and injection of healthy RGCs into a diseased eye is only the first step in a long and convoluted process of trying to reestablish the cellular connections needed to process visual information. Successful implantation would require healthy RGCs, accurate integration into retinal circuits, and axonal connections with appropriate areas in the brain. Injection of RGCs into the vitreous only results in less than 10% incorporation into the ganglion cell layer [58]. The inner limiting membrane (ILM) formed by Müller glia and astrocytes prevents facile integration both in vivo and in vitro [57, 59]. Disruption by enzymatic digestion in explants improved dispersion of transplanted RGCs with a marked increase of neurite extension into retinal parenchyma [60]. Intravitreal injections themselves cause a localized inflammatory response, which may inhibit RGC survival. Once incorporated into the ganglion cell layer, the implanted RGCs need to form meaningful synaptic connections with other cells of the inner retina and produce an axon that can reach visual targets in the brain.

In terms of building new RGCs, there have been significant breakthroughs in the effort to generate pluripotent stems cells in recent years, including the developments of inducible pluripotent stem cells (iPSCs) from human fibroblasts [61]. These cells have an advantage over embryonic stem cells since they can be generated on a patient-specific basis, limiting the possibility of an unwanted immune response. Organoids derived from pluripotent stem-cells are self-organizing three-dimensional structures of cellular networks that form in vitro after supplementation with various growth and differentiation factors [62]. Retinal organoid cups recapitulate many of the aspects of vertebrate retinal development, including gene expression and retinal lamination [63–66]. Organoid-derived RGCs also exhibit photoreceptor-driven action potentials comparable to the earliest light responses recorded from the neonatal mouse retina [67]. Planar-derived RGCs are transdifferentiated from pluripotent stem cells in culture [68], and after purification in culture have similar electrophysiological properties to native RGCs; hPSC-RGCs demonstrate characteristic spontaneous and current-evoked activity, indicating functional axons; however, they do not develop the ability to form circuitry as RGCs in organoids do [69–71]. Co-culture of hPSC-RGCs with other cell types improves dendritic complexity and electrophysiological responses [72].

One major limitation to using iPSC-derived RGCs for transplantation is that generated cells will carry the same genetic susceptibility to degeneration as the donor. For example, iPSC-RGCs derived from a donor carrying a mutation in the optineuron gene implicated in normal-tension glaucoma will carry the same gene [37, 73]. Generation of RGCs for transplantation may appear facile after discussion of the progress that has been made in the in vitro generation of RGCs; however, the retina harbors multiple retinal subtypes, each with distinct roles in the generation of visual signals [74]. Identifying the retinal subtypes that are most vulnerable to stress and degeneration in glaucoma is still challenging [14, 75]. Understanding how RGC cell types react to stress during progression will allow targeted production of specific types of RGCs, with better functional outcomes after transplantation and perhaps greater ability to produce axons capable of relaying signals to appropriate brain targets.

## Extrinsic Factors in RGC Axon Regeneration

Mature retinal tissue is an extension of the central nervous system (CNS), which has a very low capacity to regenerate after injury due to both cell-intrinsic and cell-extrinsic factors. Following optic nerve crush, axons with modest sprouting may not extend sufficiently long distances and eventually die [76]. RGCs are surrounded by a complex network of supporting cells that promotes RGC function and survival. Even so, microglia and astrocytes in the optic nerve projection produce an early neuroprotective response following injury that can impede axon regeneration [77, 78]. Microglia are immediate responders to injury, where they become reactive and secrete TNF-a, Il-1a, and C1q that recruit astrocytes to the lesion site [79]. Astrocyte reactivity and proliferation are controlled by the signal transducer and activator of transcription protein-3 (STAT3), which is a member of the JAK/STAT signaling pathway [80]. STAT3 promotes glial scar formation from newly proliferated and activated astrocytes [81]. Astrocytes remodel and increase accumulations of neurofilaments and organelles and upregulate cell-cell communication pathways to recruit metabolic resources to the site of injury [4, 82, 83••]. In spinal cord injury, glial scar formation may be initially neuroprotective by shielding neighboring uninjured axons from pro-apoptotic factors released by injured axons [84]. The removal of proliferating reactive astrocytes or genetic ablation of STAT3 accelerates axon degeneration [85]. Early inflammatory events in the retina can trigger axon regeneration; induction of inflammation by injection of zymosan into the eye or inflammation as a result of lens injury is sufficient to cause RGCs to regenerate axons through the injured optic nerve [86, 87]. Likewise, macrophage secretion of oncomodulin protein appears to promote intraocular inflammation and enhance optic nerve regeneration [88–90]. If the capacity of the retina to trigger an inflammatory response is reduced, i.e., by genetic deletion of two receptors that are expressed by inflammatory cells, Toll-like receptor 2 (TLR2) and dectin-1, then pro-regenerative effects of Zymosan injection are negated [91].

While early inflammation may benefit optic nerve regeneration, long-term glial scarring and inflammation inhibit axon growth. Reactive astrocytes and oligodendrocytes upregulate genes that inhibit axon regeneration directly or indirectly through activation of receptors on the surface of oligodendrocytes. These factors include chondroitin sulfate proteoglycans (CSPGs), Slit2, and ephrins [79, 92–94]. In healthy optic nerve, oligodendrocytes are myelinating glial cells that deposit myelin to help axon signal conductance; however, under pathogenic conditions, oligodendrocytes present a number of proteins that inhibit axon outgrowth including myelin-associated glycoprotein, oligodendrocyte myelin glycoprotein, and signaling through nogo receptors (NgR), [95–99]. Genetic deletion of NgR subtypes breaks this transduction pathway and enhances axon regrowth and motor activity after spinal cord injury; however, the results are subtle [100–102].

Late in progression, astrocyte processes fill in optic nerve volumes as glial scaring takes the place of degenerated axons [103]. Pioneering research has highlighted a far earlier role for astrocytes in providing support to injured RGC axons via metabolite redistribution in the optic nerve projection [83••]. Astrocytes are a known cellular support system for RGCs and their axons in the optic projection. Astrocytes respond to injury in the optic nerve by redistributing metabolic resources from healthy tissue via connexin-43 gap junctions [83••]. This study brings to light a novel mechanism by which astrocytes respond to axonal stress by providing auxiliary metabolic support, that if harnessed, could promote RGC survival and regeneration. Calcium-dependent astrocyte remodeling could allow focal redistribution of resources where needed [82, 104].

### Cell-Intrinsic Factors That Influence Regeneration

During early development, RGC axon growth proceeds at a rapid rate to allow the extension of axons from the retina to reach distant targets in the brain. However, as RGCs transition from an embryonic to postnatal state, they reduce their axon growth speed by over 1000-fold [39]. External growth-promoting factors in the local RGC environment are the primary driving force for axon extension during RGC maturation but attempts to capture this process through exogenous growth factors after injury have been unsuccessful [105]. A confounding factor in exogenous growth factor application is that mature neurons show decreased responsiveness to growth factors and even more so after injury [106]. Control of axon growth is likely a complex interplay between extracellular signals and intrinsic RGC signaling pathways. If such intrinsic cellular pathways are identified and harnessed, progress could be made in encouraging RGCs to grow axons post-injury.

Targeting cell-intrinsic pathways to reprogram RGCs to a proaxogenic state have been met with more success. Several intrinsic molecular pathways can influence axonal growth including members of the Kruppel-like-factor transcription family. When overexpressed, Klf-4 and Klf-9 suppress axon outgrowth [107], while overexpression of Klf-6 and Klf-7 enhances axon outgrowth. After optic nerve injury in mice, conditional knock-down or deletion of Klf-4 promotes optic nerve regeneration [107, 108]. A key molecular event that occurs during the maturation of RGCs is downregulated expression of phosphorylated mammalian target of rapamycin (phosphor-mTOR) [109]. Deletion of the mTOR inhibitor, phosphatase, and tensin homolog (PTEN) enhances the ability of neurons to regenerate after injury even in the absence of growth factors [110, 111]. Combining the effects of the mTOR pathway with inhibition of SOCS3, a JAK/STAT3 inhibitor, further enhances RGC regeneration [112]. The mTOR pathway is a ubiquitous pathway that promotes the growth and survival of cells by regulating protein synthesis [113] and appears to act as a key regulator of optic nerve regeneration [114•]. Although approaches to harness intracellular pathways seem to promote long-distance RGC axon growth, a number of hurdles remain. Some therapies, such as targeting mTOR, thus far only favor axon regeneration of a subset of RGCs [115], which would have limited benefits in patients. Other avenues may harness activity-generating cation channels that may serve to boost RGC excitation to promote survival and growth [116].

### Axon Guidance and Synapse Formation

Prompting axon regeneration from mature RGCs is a challenge in itself; once axon generation is initiated, the next hurdle is the correct direction of axon growth and generation of new synapses with targets in the brain. Efforts to regenerate axons have already demonstrated aberrant growth to improper targets [112, 117, 118]. Techniques using electric fields to direct axons to their targets have showed promise in vitro [119] and in vivo using nerve transection models [120–122], but safer application of electrical fields will determine whether this technique can be applied to humans [123]. An alternative approach using 3D-printed scaffolds to aid in axon guidance has been implemented in vitro [69, 124] but is in their infancy in their translation to the clinic.

As well as proper guidance to terminals, axons need to establish functional synapses in the diencephalon. Progress in this field is already hampered by limitations in long-distance axon regeneration. Going forward, understanding the process of synaptogenesis and cellular mechanisms involved in RGC development may help to promote synaptic development in regenerated RGCs. For example, in utero, developing RGCs are primed to respond to light stimulation by spontaneous neuronal activity called “retinal waves” [125]. This process drives voltage-dependent influx of calcium, which alters transcription of genes to promote synapse formation. Harnessing these developmental systems may promote synaptic formation in transplanted RGCs.

## Conclusions and Future Directions

We face many challenges in trying to restore lost vision for patients with advanced glaucoma. The main focus of next generation glaucoma therapies is in the neuroprotection of RGCs to prevent vision loss and in cell replacement therapies to restore vision. Re-wiring the visual system is not a facile goal, the first obstacle lies in identifying windows of restorative opportunity. The earliest window of opportunity is when RGCs first become dysfunctional; in animal models, this is easily identified; however, in humans, it is more challenging to identify early RGC dysfunction. Currently, elevated IOP is the only indicator that RGCs are experiencing increased stress, and it is at this point that our neuroprotective strategies would be most effective. Further research into improved retinal imaging in the clinic would greatly enhance the efficacy of neuroprotective therapy by identifying early RGC dysfunction. It is unlikely that neuroprotective strategies alone will prevent RGC loss, since glaucoma patients often progress with the disease until visual defects occur.

Cell replacement therapy has long been a tantalizing prospect for many neurodegenerative diseases. The potential therapeutic value of RGCs derived from retinal organoids and planar systems is vigorously under investigation by numerous groups facilitated by the National Eye Institute’s “Audacious Goals Initiate for Regenerative Medicine.” There are still hurdles to overcome however; for example, defining the most effective delivery system for donor cells into the host retina, examining how the introduction of foreign cells impacts the native retinal environment, and determining if donor RGCs integrate and form functional circuits that restore visual processing. The restoration of vision requires transplanted RGCs to grow axons that extend beyond the optic nerve head, remyelination by oligodendrocytes, and correct guidance to terminals in the brain where new synaptic formation can occur. RGCs do not function in isolation, relying on both cell intrinsic and extrinsic factors during disease to determine cell fate. In fact, RGCs rely on multiple cell types for survival in the optic tract. The novel finding that astrocytes directly act to provide neuroprotection during stress by shuttling metabolic resources further drives home this fact. Therefore, future progress in vision restoration will lie in harnessing the support mechanisms by other cells in the visual system to promote effective RGC transplantation and the formation of functional visual circuits.
